# Objections and Difficulties

**Published:** 1916-02-26

**Authors:** 


### OBJECTIONS AND DIFFICULTIES.

I will now refer to certain objections and difficulties. In the first place, it is said that this work is not a suitable form of employment for women. In reply to this it may be pointed out that the work in question is actually being done by women in all but two of the Scottish asylums, and the consensus of opinion in Scotland, where experience of the system is unsurpassed, not only does not support, but contradicts the objection. The personal offices that all nurses, including hospital nurses, may be called upon to perform in the nursing of adult males may be objected to and have been objected to. Between fifty and sixty years ago, before the movement started by Florence Nightingale had been given time to effect a reformation, I have been informed by my teachers that no woman with any self-respect or regard for her reputation nursed adult males in our general hospitals. Women from every rank of society are prepared to do so now, and are held in the highest esteem if they do. Is is not, then, clear that it is not the work alone that matters, but that the spirit in which it is done, the methods that are employed, and the character of the person who comes to the work, are essential elements in any judgment upon it. If, therefore, the status of hospital nursing can be so transformed in the estimation in which it is held, may not a similar change by the adoption of similar methods take place in connection with asylum nursing? If approached in the proper spirit, if performed by approved methods, and if undertaken by the right persons, this form of employment has been found in Scotland quite suitable for women.

In introducing female nurses into the male wards for the first time the most reliable women on the staff would naturally be selected by anyone who wished the experiment to be a success. They should be experienced and they should not be young. The working unit should not be less than four in number. It is a great advantage to place a hospital nurse with asylum experience in charge of them. The methods employed in handling the patients and in the management of the ward should be those which have been adopted in general hospitals, on account of their regard for the decencies. It is naturally found that these can be most readily adopted for those patients who are confined to bed. When the patients are dressed and going about it is advisable to employ auxiliary male care in the form of one or two trustworthy married attendants to bathe the patients and to assist in other ways if required. I have never had the least difficulty in arranging for this small amount of auxiliary male care, and I have always found convalescent and working male patients willing to help the nurses.

In the second place, it has been said that the male side of an asylum is not a fit place for a woman to be in. The presence of good women always has a refining influence on male society, and whatever the conduct of male patients in speech and in general behaviour may be, the advent of female nurses among them, if managed with care, will effect a change for the better. The capacity of the insane for education in good habits, while not illimitable, is very extensive, and in practice it is never exhausted in our large institutions. Were we not so familiar with it, their behaviour and self-control, for example during Divine service, would astonish us every week as it does those who see it for the first time. If, therefore, it can be alleged of any asylum that its male wards are not a suitable place for women, then the sooner a reformation be effected the better for the patients there, for it is not a condition that need continue indefinitely.

It is then asked, Are women who object to nurse male patients to be compelled to do this work? The answer is, Of course not. There are women who object to nurse male patients just as there are women who object to be nurses at all; but of the hundreds who have been nurses in the asylums of which I had charge those who have objected during twenty years can be counted on the fingers of one hand. As a matter of fact, the vast majority prefer to do so, and the reason is not difficult to find. Male patients are always less troublesome and excitable than female, and women find that they receive more courtesy and readier obedience from men than from members of their own sex. They do not require to receive any extra salary to do this work once it has been started, for the women are engaged, as they are in general hospitals, simply to nurse, and it is all in the day's work whether they nurse patients of the male sex or of the female. It is very doubtful if there be any saving in expenses by the employment of women instead of men, because, owing to the higher standard of hospital care aspired to, there is usually found to be a larger number of nurses required. In Scotland, any saving there may have been from this source has been more than expended on an increased night staff, which is proportionally much larger than that employed in English asylums, and on hospital nurses for purposes of supervision, which is a practice that has now been largely adopted.

Lastly, it has been pointed out that many male patients, owing to their sexual proclivities, cannot be cared for by women. This is undoubtedly true, but the remedy is a simple one : do not place them under the care of women; let them be cared for by men. It is unthinkable that any experienced administrator would allow a simple difficulty of this kind, with an obvious remedy, to deter him from the introduction of women nurses. It may give him a little more trouble, which at present he escapes, but that is no excuse for avoiding a duty. Every day of the year, in every asylum in the country, a much more difficult and responsible task of an analogous nature is faithfully performed, that of distinguishing the patients who are suicidal from those who are not, and of making special arrangements for their care. To pick out patients whom it is undesirable to place under the care of women is, compared with this, an exceedingly simple matter.

Far from the employment of female nurses in the male wards of asylums being unsuitable in form, out of place, and objectionable to them, in the high state of organisation and development now attained by mental hospitals, whatever may have been the case in the past, it is most appropriate and a beneficent duty to the insane male patients under our care. They appear to be the last class of the helpless to benefit from the superior aptitude and skill that women show for the duties of nursing, and this privilege should no longer be denied them, as it is overdue. The reason for this superiority of female nursing rests on a solid foundation, the mothering instinct in women. It is an instinct so strong that in many cases it cannot be suppressed and must manifest itself in one form or another. There are, of course, exceptional women and exceptional men, and we have all met male attendants who have been kind and devoted nurses. Nevertheless, nursing the sick, the infirm, and the helpless, be they sane or insane, is pre-eminently woman's avocation. Sir Thomas Clouston summed up the situation tersely when he said that all his nurses longed to work in the hospital, whereas all his male attendants wished to be kept out of it and preferred to do outdoor work, and that he never saw a man enjoy sick-nursing in the same way as many women do.

It has been remarked that only a small proportion of the male patients are in the hospital wards, but is not a great part of the work in an asylum indoor domestic duties, which in a private house are also performed by women? The cleaning and decoration of the wards, the bedmaking, the laundry, and repair of the clothing, the serving of the food, and the social functions are all tasks which in private life usually fall to the lot of women. Can it, then, be doubted that they are as efficient as, if not more so than, men, to perform these familiar occupations in asylums?

Moreover, it must not be assumed that female nurses are only of use for the care of the sick and helpless in an asylum. One of the surprises of the system in practice has been the discovery that they can usually exercise more control over cases of mania than male attendants, and the great advantage of their management lies in this fact, that it is based on persuasion and not at all on the show of force or on compulsion. Excited patients who are ready to fight any man who comes near them will often do anything they are told by a nurse, and they will become calm if they receive a word of sympathy from her. A woman has much the same influence over an insane man, who is not actually delirious, as she has over one who is supposed to be in his sound mind, and it is absurd to assume that all feelings of chivalry and honour die in a man because he suffers from some derangement of the mind.

### The Proportion of Women.

The proportion of women it is desirable to employ on the male side of an asylum is, according to the Scottish Board of Control, at least 25 per cent. of the total day staff on the male side and 15 per cent. of the night staff. These figures are considerably exceeded in several asylums, among which may be mentioned the Stirling District Asylum. It may be taken as a typical county asylum in the accommodation it provides, in its complete organisation, and in the modern methods it employs. It admits over 250 patients annually, and has a resident population of over 800. Dr. R. B. Campbell, its medical superintendent, has employed for the last seven and a-half years—as I also did for an equal period—a staff on the male side by day of which 40 per cent. consists of women, there being three hospital nurses, including the matron. By night 27 per cent. of the staff consists of women, including the night superintendent, who is a trained hospital nurse.

### Parochial and Private Patents.

It has been stated that female nurses are more suitable for asylums admitting parochial than private patients of the richer classes. That has not been my experience at Craig House. This is the department of the Royal Edinburgh Asylum for private patients, and it is quite a separate mental hospital from the West House, which provides accommodation for poorer patients. Of the staff of thirty-two employed on the male side by day to attend to 100 gentlemen, exactly one-half consists of nurses, including in this number the lady superintendent and three matrons, and of these three are hospital nurses. By night, six out of a staff of thirteen consist of women, including the night superintendent, who is a trained hospital nurse. These proportions vary from day to day, acording to requirements, and they have perhaps been swelled by the war, but there is no difficulty in employing 40 per cent. of women by day and 25 per cent. by night in a private asylum like Craig House. Of course there are special difficulties connected with private male patients which are not met with in the case of parochial patients, but the employment of women in their care on the whole is equally advantageous. The opinion of the friends of patients is worth quoting. The most interested relatives of gentlemen consist chiefly of anxious females, be they mothers, wives, or sisters, and nothing, in my experience, gives them greater comfort than to know that the relatives whom they entrust to our care will be tended by women. Rightly or wrongly, to them it is a guarantee that no violence will be employed, and that the most skilled nursing will be available.

### Conclusion.

In conclusion, I have to state that these opinions, whatever may be their value, are founded on twenty years' experience of entire female nursing in male wards, and have been gained in four different asylums of which I have had charge during that time. I am now more convinced than ever that the mental hospital, the modern asylum, is only a hospital for the treatment of a special disease, and therefore requires to be run on hospital lines, of which the employment of women in the male wards is only one feature. Although many did not see eye to eye with me in the past, I have learned to be patient, and I have had the satisfaction of seeing these views gradually accepted and the methods I advocated adopted. The ultimate hospitalisation of the asylum is now only a question of time, and that time has been hastened by the action of many medical superintendents of the English asylums, who, owing to one of the results of the war, have introduced female nursing in the male wards of their asylums for the first time. I trust that the observations I have made may assist others in coming to a similar decision.

